# Nonporous, Strong, Stretchable, and Transparent Electrospun Aromatic Polyurea Nanocomposites as Potential Anticorrosion Coating Films

**DOI:** 10.3390/nano11112998

**Published:** 2021-11-08

**Authors:** Sheik Ambarine Banon Auckloo, Khanisya Palaniandy, Yew Mun Hung, Giuseppe Lazzara, Siang-Piao Chai, Pooria Pasbakhsh

**Affiliations:** 1 Mechanical Engineering Discipline, School of Engineering, Monash University Malaysia, Jalan Lagoon Selatan, Bandar Sunway, Subang Jaya 47500, Malaysia; sheik.auckloo@monash.edu (S.A.B.A.); khanisya.palaniandy1@monash.edu (K.P.); hung.yew.mun@monash.edu (Y.M.H.); 2 Department of Physics and Chemistry, University of Palermo, Viale delle Scienze, pad. 17, 90128 Palermo, Italy; giuseppe.lazzara@unipa.it; 3 Multidisciplinary Platform of Advanced Engineering, Chemical Engineering Discipline, School of Engineering, Monash University Malaysia, Jalan Lagoon Selatan, Bandar Sunway, Subang Jaya 47500, Malaysia; chai.siang.piao@monash.edu

**Keywords:** polyurea, nanocomposites, thin film, coatings, electrospinning, deposition

## Abstract

This study, for the first time, focused on the fabrication of nonporous polyurea thin films (~200 microns) using the electrospinning method as a novel approach for coating applications. Multi-walled carbon nanotubes (MWCNTs) and hydrophilic-fumed nanosilica (HFNS) were added separately into electrospun polyurea films as nano-reinforcing fillers for the enhancement of properties. Neat polyurea films demonstrated a tensile strength of 14 MPa with an elongation of 360%. At a loading of 0.2% of MWCNTs, the highest tensile strength of 21 MPa and elongation of 402% were obtained, while the water contact angle remained almost unchanged (89°). Surface morphology analysis indicated that the production of polyurea fibers during electrospinning bonded together upon curing, leading to a nonporous film. Neat polyurea exhibited high thermal resistance with a degradation temperature of 380 °C. Upon reinforcement with 0.2% of MWCNTs and 0.4% of HFNS, it increased by ~7 °C. The storage modulus increased by 42 MPa with the addition of 0.2% of MWCNTs, implying a superior viscoelasticity of polyurea nanocomposite films. The results were benchmarked with anti-corrosive polymer coatings from the literature, revealing that the production of nonporous polyurea coatings with robust strength, elasticity, and thermal properties was achieved. Electrospun polyurea coatings are promising candidates as flexible anti-corrosive coatings for heat exchanges and electrical wires.

## 1. Introduction

Metals are vastly used in myriad applications such as aerospace, aviation, automotives, electronics, or oil and gas. However, they are highly exposed to environmental aspects such as high salinity, water, oxygen, high pH, or aggressive conditions that often lead to their rusting and corrosion [1,2]. Organic coatings act as barriers, corrosion inhibitors, and electrochemical protectors, protecting the metals from getting into contact with the external environment and decreasing the rate of corrosion. Organic coatings such as polymeric coatings used as a top-coat for anti-corrosion demands properties such as hydrophobicity, durability, good substrate adhesion, flexibility, high thermal resistance, strength, and easy application [3]. These coatings protect metals from corrosion via different mechanisms such as the barrier effect, inhibitive effect, or galvanic effect if metallic fillers such as zinc are used as a sacrificial anode. The barrier effect delays the diffusion of water, ions, or oxygen to and from the metal surface due to the low permeability factor [4]. The inhibitive effect, on the other hand, is due to the coatings’ high electrical resistivity, which prevents ionic current from flowing into the metallic substrate [3].

Polymer composites are one of the materials used to prevent the corrosion of metals. Polyurethane [5,6,7], epoxy [8,9,10], polydimethylsiloxane (PDMS) [11,12], polyaniline [13,14], polythiophene [15,16], polypyrrole [17,18], polyurea [19,20], and high-density polyethylene [21] are some of the commonly used base polymers for coatings. The incorporation of fillers such as graphene oxide [22,23,24], multi-walled carbon nanotubes (MWCNTs) [25,26], zinc oxides [27,28], silicon oxide [29,30], or titanium oxide [31] in the polymer matrix has been shown to reduce the corrosion rate of metals in the past. The addition of MWCNTs may lead to an increase in mechanical strength, thermal stability, and hydrophobicity, therefore acting as a barrier for the passing of electrolytes [26]. Moreover, MWCNTs can act as UV absorbers, thus reducing the degradation of the coating. On the other hand, silicon oxide (SiO_2_) is known to create a physical barrier and prevent the metal surface from anodizing [32].

Asmatulu et al. [33] studied the effect of UV degradation on epoxy with MWCNTs loaded at increasing weight ratios. It was found that MWCNTs reduced the percentage thickness loss and cracks upon exposure to UV and salt fog [33]. The addition of MWCNTs reduced the crack formation, therefore limiting the access of corrosive substances to the metal surface, reducing corrosion. Similarly, a poly(3-aminobenzoic acid)-MWCNT nanocomposite coating on copper improved the corrosion inhibition efficiency, aiding the barrier effect, whereby the corrosion current density and the corrosion potential were 0.562 μA cm^−2^ and −103 mV, respectively [34]. Moreover, in a study by Jeon et al. [35], MWCNTs were shown to increase the hydrophobicity of the polymer coatings, retard water penetration, and reduce the saturation capacitance of water. This phenomenon is due to two mechanisms: (1) the coatings’ hydrophobicity repels water molecules from its surface and (2) MWCNT nanoparticles fill the pores or spaces left by the crosslinking of polymer chains, preventing the penetration of corrosion media such as water [35]. Alternatively, silica nanoparticles are rheological modifiers, which increase the coating’s viscosity, and as a result, prevent the flow around sharp corners [36]. This increases the stability and thickness of the coatings on the metal surfaces, therefore enhancing corrosion protection. Bakhtiary-Noodeh et al. [36] studied the effect of hydrophilic nanosilica in a commercial waterborne automotive electrocoat (BASF) coating on steel. It was found that at an optimal loading of silica (up to 6%), the anti-corrosion effect was enhanced; however, at a loading of 8%, it drastically decreased [36]. It is understood that the presence of MWCNTs and nanosilica increases the barrier resistance due to better packing, making the path for corrosive electrolytes lengthier and complex. Nevertheless, at high percentage, there could be inappropriate dispersion, leading to a loss of resistivity of the coating or lack of adhesion to the metal surface [36,37].

The deposition method of anti-corrosive coatings is a crucial factor in determining the surface morphology. Coatings are usually produced by spin casting [38,39,40], molecular-layer deposition (MLD) [41,42], vapor-deposition polymerization (VDP) [43,44], or spray coating techniques [45,46]. These methods adopt a layer-by-layer deposition approach, and each layer adheres to each other rapidly, hindering the crosslinking of polymer chains and resulting in cracks and low strength [47,48,49,50]. In order to prevent nucleation growth during MLD, self-assembled precursors are added; however, they also diffuse easily into the porous deposited films [49]. This process leads to additional film growth and affects the controllability of the MLD process [51]. In addition, MLD is highly dependent on the substrates, precursors, and growth per cycle, making the process tedious, and requires a high-energy consumption [52]. MLD, VDP, and spin casting also require expensive equipment and are difficult to apply to large surface areas and irregular geometries [32]. Spray coating, in contrast, deposits microdroplets, leading to the formation of pinhole defects undetectable to the naked eye [53], therefore allowing oxygen and chloride ions to attain the metal and consequently accelerating the corrosion process. On the other hand, electrospinning has been used to coat polymer fibers onto metal surfaces, and it was shown to decrease corrosion greatly due to the high adhesive strength achieved [54,55,56]. While fibers produced by electrospinning could delay corrosion, the produced micropores create an influx of corrosive substance to the metal surface. For example, Zhao et al. [57] developed a bi-layer coating consisting of electrospun polyaniline (PANI)/polymethylmethacrylate (PMMA) and a sprayed topcoat of polystyrene. The polystyrene served as a protective barrier, while the PANI/PMMA acted as an anodic protection, showing an efficiency of 99.95% when the topcoat was added compared to 92.06% with only electro-spun PMMA/PANI [57]. The electrospinning technique caters for the irregular size and complex geometries of metal surfaces; however, due to the porous structure of the film, it was never an ideal solution to create a standalone nonporous coating. The extra multilayered coating to cover the base porous structure leads to excessive material and production cost for industrial applications. Electrospinning of a nonporous thin polymer has remained a huge challenge in both research and industry due to the controllability of many factors at the same time such as polymer viscosity, molecular weight, solvent concentration, and distance and potential difference between the nozzle and the surface [58].

Elastomeric polymers such as polyurea or polyurethane demonstrate superior strength and elasticity due to their segmented structure and viscoelasticity; thus, they could be good candidates as nonporous anti-corrosive coatings. These elastomers have a hard segment (HS) and soft segment (SS) that caters to their strength and elongation. Polyurea is a mechanically robust polymer derived from a step-growth polymerization of diisocyanate (component A) and diamine polymer chains (component B). The micro-segmented structure is due to the thermodynamic incompatibility of both HS and SS [59]. Polyurea demonstrates elastomeric properties due to polyurea chains’ crosslinking and high elongation properties [60,61,62]. It is known to have higher elongation, and many studies have reported it to be in the range of 300–1000% when cast or spray coated. It exists as aromatic and aliphatic (or a blend of two) depending on the isocyanates used, where aromatic is known to be less orderly packed than aliphatic due to the presence of the benzene rings in aromatic isocyanate [63]. The micro-phase separation in polyurea exists as soft domains and hard domains induced by bidentate hydrogen bonding between the hard segments. The phase separation is of a higher degree with aromatic hard segments due to the increased thermodynamic inconsistency between aliphatic SS and aromatic HS, resulting in higher strength [64]. The exceptional mechanical strength, flexibility, and micro-phase structure of polyurea make it a great candidate as an anti-corrosive coating for metal substrates. Fabricating polyurea as a nonporous film will reduce the penetration of corrosive substances due to the complex micro-phase separation and cross-linking of the polyurea chains.

Studies on the electrospinning of polyurea are scarce to the best of our knowledge. The latest study by Tripathi et al. [65] demonstrated the production of nonwoven fibrous electrospun aromatic polyurea membranes with a low-molecular-weight amine at a concentration of 30% by weight of polyurea in N,N-dimethyl formamide (DMF). The resulting tensile strength was 15 MPa but with an extension of around 5%. The flexibility obtained may be limited due to the short amine chains, resulting in a low percentage of the soft segment. These thin polyurea film production complexities motivate the need for high-strength, stretchable, and optically transparent nonporous polyurea films as coatings and protective barriers.

This paper focused on producing solid (nonporous) thin-film polyurea nanocomposites using the electrospinning technique. MWCNTs and hydrophilic fumed nanosilica (HFNS) were incorporated at increasing loadings to show the effects of the nanoparticles on the mechanical strength, hydrophobicity, and thermal stability of nonporous electrospun polyurea films. The properties of fabricated polyurea/MWCNT and polyurea/HFNS nanocomposites were characterized using dynamic, mechanical, tensile, thermal, physical, and morphology analysis tests. Our novel approach to produce thin nonporous films by electrospinning is useful in applications including anti-corrosive layers for metallic substrates or electronics containing corrosive materials.

## 2. Materials and Methods

### 2.1. Materials

Polycarbodiimide-modified diphenylmethane diisocyanate was purchased from Pacific Urethanes (Victoria, Australia). Oligomeric diamine (Versalink P650) was purchased from Finn Chemicals Sdn Bhd (Petaling Jaya, Malaysia. Aerosil 200, a hydrophilic fumed nanosilica (HFNS) produced by Evonik Industries, was purchased from Johnson & Johnson Sdn Bhd (Petaling Jaya, Malaysia). Multi-walled carbon nanotubes (MWCNTs) were purchased from Advanced Nano Powder Inc. (Taiwan). Organic solvent N,N-dimethylformamide (DMF) was purchased from Fischer Scientific (Shah Alam, Malaysia). All materials were used without any further treatment.

### 2.2. Solution Preparation and Experimental Setup

**Figure 1:**
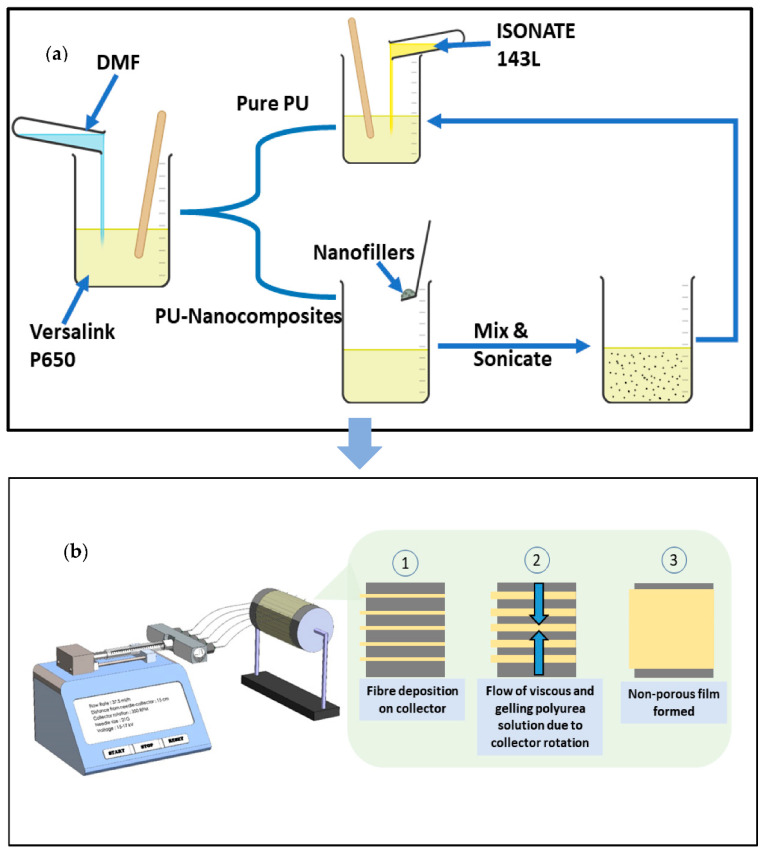
Process of preparation of polyurea nanocomposites thin films. (**a**) Solution preparation; (**b**) electrospinning process.

The preparation process is demonstrated in Figure 1a. First, 50 wt.% of DMF relative to the mass of polyurea (PU) was added to component B. The solution was mixed thoroughly until a homogenous solution was obtained. In the case of neat PU solution, component A was added and mixed simultaneously. For the nanocomposite solution, the nanomaterials (MWCNTs and HFNS) were added to component B at different wt.% (0.2%, 0.4%, 0.6%, and 1%), and the mixture was sonicated for 10 min at an amplitude of 50 Hz before addition of component A. The samples in this study were referred to as PU-X%MWCNT for the sample with a X% MWCNT loading and PU-X%HFNS for the sample with a X% HFNS loading. In both cases, the final solution was degassed at -1 bar in a vacuum oven at room temperature to break any air cavitation present in the solution. The solution was poured into a 20 mL Terumo syringe and attached to a syringe pump. The solution was fed to a five-nozzle linear spinneret with 21G flat Agani disposable needles. The flow rate was set at 37.5 mL/h (7.5 mL/h per nozzle). The critical voltage was achieved at the point of appearance of a constant and stable Taylor’s cone. The electrospinning was carried out on a rotating collector at a speed of 350 RPM with aluminum foil as the substrate. The complete setup is shown in Figure 1b. In the first stage, the fibers were deposited on the collector, with the continuous rotation of the collection and the gelling state of polyurea, and the fibers flowed and merged, creating a nonporous film. The final film was left to cure for 24 h before being removed from the foil with ethanol. The samples were post-cured in the oven at 80 °C for 24 h before testing was carried out.

### 2.3. Characterization

#### 2.3.1. Tensile Testing

The samples were cut to a size of 15 mm × 100 mm (Figure S1 in Supplementary Materials) and double-sided foam adhesives of 25 mm × 20 mm were placed at each end to create a tab in between the clamps. The samples were then tested according to ASTM D882 at a grip separation rate of 500 mm/min. The thickness was measured using a Mitutuyo high-accuracy digital micrometer of accuracy +/− 0.0001 mm. The films were placed between two glass slides. Three measurements were taken, the average thickness was obtained, and the glass slides’ thickness was subtracted. The average thickness of the film was used during testing. Three films for each composition were tested, and the statistical mean and standard deviation of tensile strength and elongation at break were computed using GraphPad Prism software (version 9). Dunnett’s multiple comparison tests using one-way ANOVA were performed to compare the mean of the samples to the mean of the neat PU with a 95% confidence (*p* < 0.05 is indicated as significant in the discussion).

#### 2.3.2. Fourier-Transform Infrared (FTIR)

Fourier-transform infrared (FTIR) was carried out to determine the chemical bonds of each sample after post-curing to ensure complete polymerization using a FTIR spectrophotometer (Nicolet iS10, Thermo Scientific, Waltham, MA, USA) in the range from 500 to 4000 cm^−1^. The sample was cut into a square shape of 5 mm in length. A total of 64 scans were performed for absorbance vs. wavenumber.

#### 2.3.3. Water Contact Angle

The degree of hydrophobicity was measured in a laboratory setup using a smartphone with a 15X macro lens, 0.1059 mm needle, and a 10 mL Terumo syringe purchased from SJ Surgical Supplies (Terumo, Subang Jaya, Malaysia). The sample was placed on a flat metal plate and was fixed using a retort stand. A droplet of water was placed onto the sample using the needle, and pictures were taken at intervals of 30 s for 4.5 min. The water droplets were analyzed from the ImageJ software (version 1.53c) using the LBDSA plugin by DROPAnalysis [66].

#### 2.3.4. Contact Transparency

The transparency test was performed by placing a 1 cm × 2 cm sample on a white paper with MONASH writing, and pictures were taken in ambient light.

#### 2.3.5. Field Emission Scanning Electron Microscope (FE-SEM)

The surface morphology was studied using a Field Emission Scanning Electron microscope (FE-SEM) (Hitachi SU-8010, Hitachi, Tokyo, Japan) by placing the sample on a sample holder with double-sided black tape. It was then coated with gold particles to enhance the conductivity. The surface morphology was then studied under FE-SEM using low and high magnification. The voltage used ranged from 2 to 5 kV.

#### 2.3.6. Modulated Thermogravimetry (MTGA)

A thermogravimetric (GA 550 (Discovery series), TA Instrument, New Castle, DE, USA) apparatus was used to measure the thermoanalytical curves under a nitrogen flow. Each sample (ca. 10 mg) was heated up with a modulated temperature ramp consisting of an average heating rate of 2 °C min^−1^ and an oscillation amplitude of 5 °C with an oscillating period of 200 s. From these experiments, the degradation temperature was taken at the maximum of the first-order derivative curves of mass loss to temperature (DTG curves) for the first (T_d1_) and second degradation steps (T_d2_).

#### 2.3.7. Dynamic Mechanical Analysis (DMA)

Dynamic mechanical analyses (DMAs) were carried out on the polyurea composite films with a rectangular shape (10.00 × 5.00 × 0. 200 mm^3^) by using a DMA Q800 apparatus (TA Instruments, New Castle, DE, USA). The experiments were performed through a tensile clamp in the oscillatory regime (frequency of 1.0 Hz and stress amplitude of 0.2 MPa) by heating the sample from 25 to 170 °C. The heating rate was set at 4 °C min^−1^. DMA measurements allowed us to estimate the viscoelastic response of the films to the variations in the temperature.

## 3. Results and Discussion

### 3.1. Thickness and Transparency of Polyurea Membranes

**Figure 2:**
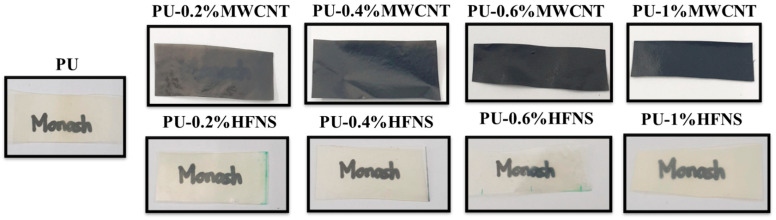
Transparency of polyurea and polyurea nanocomposites thin films.

The polyurea films’ thickness was around 200 to 250 μm when 20 mL of the solution was spun on an aluminum sheet of 25 cm × 20 cm. The transparency of the samples is shown in Figure 2. Neat PU demonstrated a higher transparency as compared to the other composites. The high transparency for neat polyurea thin film could be due to the microphase separation as hard and soft domains being smaller than the visible light wavelength (400–800 nm) [67]. Samples with multi-walled carbon nanotubes (MWCNTs) and hydrophilic fumed nanosilica (HFNS) became less transparent when the loading of the fillers increased. This physical change could be explained by the increased light scattering within the polymer structure due to reflection and refraction of the incident light on the surface of the nano-reinforcement. Moreover, nanofiller agglomeration could also promote the scattering of light due to the sizes of agglomerates being nearer to the wavelength of light (400 nm) and the different refractive index from the medium they are in, making the samples less transparent [68,69].

### 3.2. Mechanical Properties of Polyurea Nanocomposite Membranes

**Figure 3:**
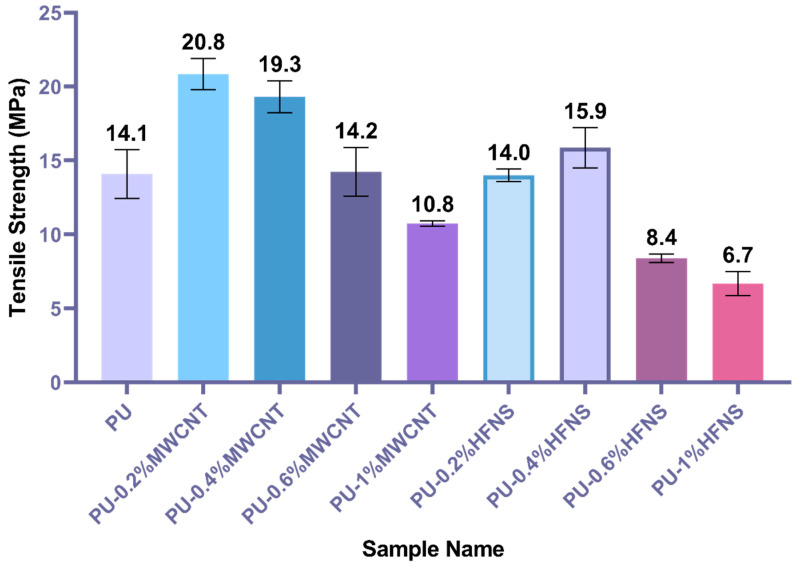
Tensile strength of polyurea and polyurea nanocomposites.

Tensile testing of the prepared electrospun neat polyurea films demonstrated an average tensile strength of 14.1 MPa with 360% elongation at break. As for the nanocomposite polyurea films (Figure 3), the tensile strength of polyurea films increased to 20.8 MPa and 19.3 MPa with the addition of 0.2% and 0.4% of MWCNTs, respectively. Statistical analysis using the one-way ANOVA with Dunnett’s multiple comparisons test of a 95% confidence interval (*p* = 0.05) (Table S1 in Supplementary Materials) was performed on the tensile strength of all the reinforced samples and compared to the pure polyurea film. The analysis further confirmed the significant increase in tensile strength after adding 0.2% and 0.4% of MWCNTs with P values of less than 0.001. Moreover, with a 1% MWCNT addition, the tensile strength significantly decreased, and this could be attributed to the nonuniform distribution, agglomeration of MWCNTs, and disruption in the hard domains due to the infiltration of MWCNTs in between the polymer chains.

On the other hand, polyurea-HFNS nanocomposite films did not improve the tensile strength, except that the 0.4% HFNS tensile strength increased slightly to 15.9 MPa. However, statistically, the increase in tensile strength was not significant with a *p*-value of 0.268, concluding that HFNS did not improve the tensile strength of polyurea film. The difference between HFNS and MWCNTs might be explained by the different structures of those two reinforcers. Polyurea is not an excellent candidate to be reinforced by nanofillers, as it is intrinsically reinforced by the hard segments [70]. Consequently, the reinforcement of polyurea highly depends on the chemical interaction between fillers and the polyurea matrix.

Moreover, the structure of the nanofillers is highly dependent on its integration within the matrix. For example, trisilanolphenyl-functionalized polyhedral oligomeric silsesquioxane (POSS), a three-dimensional cage-like structure as compared to HFNS (spherical) and MWCNTs (tubular), increases the mechanical strength of polyurea, due to its reaction with isocyanate to produce polyurea, as well as increases the cross-linking density by hydrogen bonding [70]. HFNS particles may have caused a higher agglomeration, therefore disrupting the intrinsic hydrogen bonding between polyurea chains. In contrast, MWCNTs being multi-walled thin hollow tubes would provide a larger surface area outside the tubular structure for noncovalent entanglement of the polyurea chains, creating more resistance in the breakage of the crosslinks and hard domains during force loading. However, the lowered strength at a higher loading of MWCNTs could be due to the increase in viscosity of component B of polyurea, resulting in the entrapment of air [70]. The addition of MWCNTs and HFNS at higher loading leads to agglomeration, creating a physical hindrance between the polyurea chains, such that the crosslinking and orientation of polyurea chains are disrupted, resulting in an irregularity in the packing of the polyurea chains and the inability of having a uniform distribution of the hydrogen bonding, leaving gaps and voids at some instances. This causes a disruption in the physical crosslinking and a reduction in the chains dynamics and local stress concentration, thus resulting in an early fracture.

Subsequently, the functionalization of MWCNTs can further improve (not performed in our study) the interfacial interaction of MWCNTs with polyurea. This is because the polyamine can be covalently grafted on the functionalized MWCNTs; therefore, the agglomeration of MWCNTs within the polyurea matrix will be greatly reduced and a more ordered matrix will be obtained [71].

**Figure 4:**
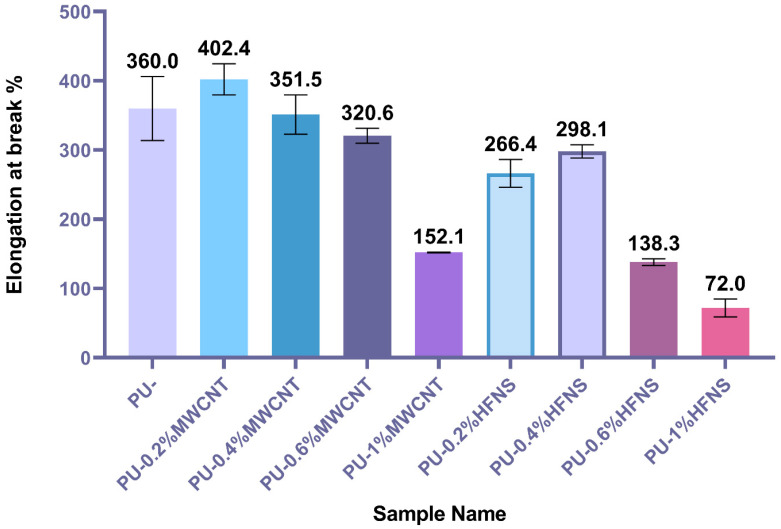
Elongation at break of polyurea and polyurea nanocomposites films.

Figure 4 demonstrates the elongation at break of polyurea and polyurea nanocomposites films. PU-0.2%MWCNT demonstrated the highest elongation at 402.4%, while neat PU demonstrated an elongation of 360%. According to ANOVA statistical analysis (Table S2), MWCNTs did not affect the elongation significantly except for PU-1%MWCNT, whereby the elongation reduced to 152.1%. At the same time, HFNS composites showed a significant decrease in comparison with neat PU. This might be explained by -OH groups surrounding the HFNS particles, which could disrupt the hard domain of polyurea chains whereby a carbonyl group from one chain crosslinks with a -NH group in other chains within the urea linkages. The -OH group in HFNS is more prone to hydrogen bonding due to the higher dipole than the -NH group, disrupting the crosslinking and reducing the strength and elongation.

**Figure 5:**
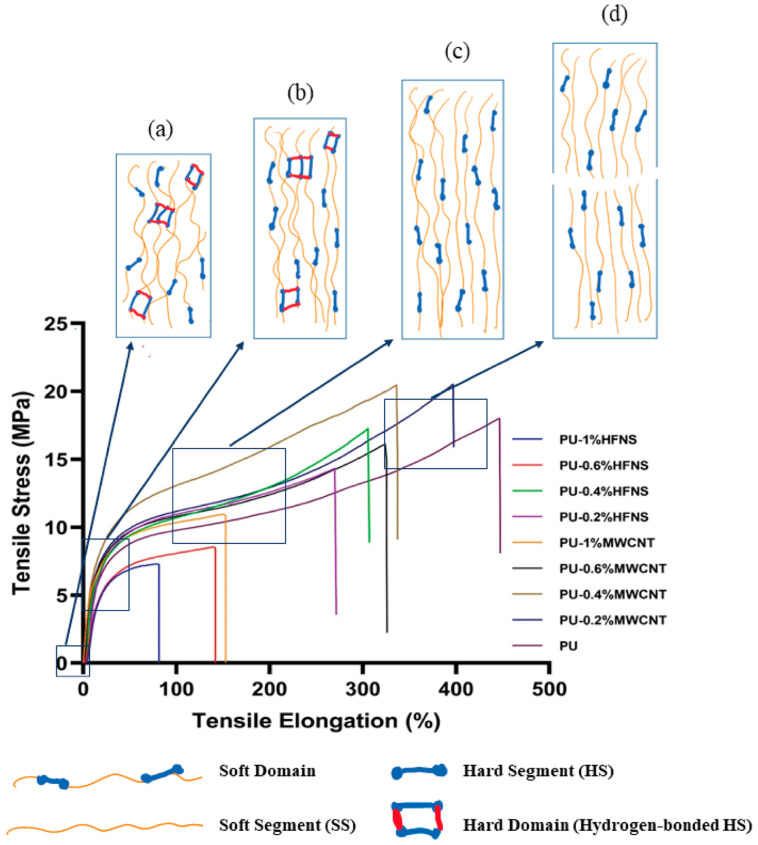
Engineering stress–strain of samples demonstrating highest tensile strength of polyurea and polyurea nanocomposite (**a**) chain arrangements at 0%, (**b**) chain motions during elastic deformation, (**c**) breakage of hard domain during plastic deformation and (**d**) fracture of polyurea chains.

The reinforcement and failure mechanism of polyurea nanocomposites are also explained in Figure 5. The elastomeric behavior of polyurea could be observed as it went through an initial linear elastic deformation in the primary loading phase. Stress was proportional to strain followed by strain hardening. The stress increased at a slower rate with an increase in strain. There was an abrupt increase in stress in the last stage until fracture, demonstrating a significant strain hardening effect. Typically, the strain hardening effect in polyurea can sustain the film for a longer time with continuous stress. The presence of hard domain aggregates can explain the strength and the elastomeric property of polyurea due to hydrogen bonding, which restricts the movement of the chains and the reordering of the soft segment upon strain [72,73].

Figure 5a demonstrates the chain alignment before loading, whereby there was an entanglement of the polyurea chains with crosslinking by hydrogen-bonded hard segments. In the first phase (Figure 5b) of the deformation, polyurea chains realigned in the direction of the force even though there was resistance as the macromolecule flowed across each other. In the second phase (Figure 5c), there was a breakdown of the hard domains as the hydrogen bonds between the hard segments were broken, resulting in only soft domains. Finally, in the last phase, as shown in Figure 5d, there was a nearly linear increase in stress until fracture.

### 3.3. Chemical Interaction

**Figure 6:**
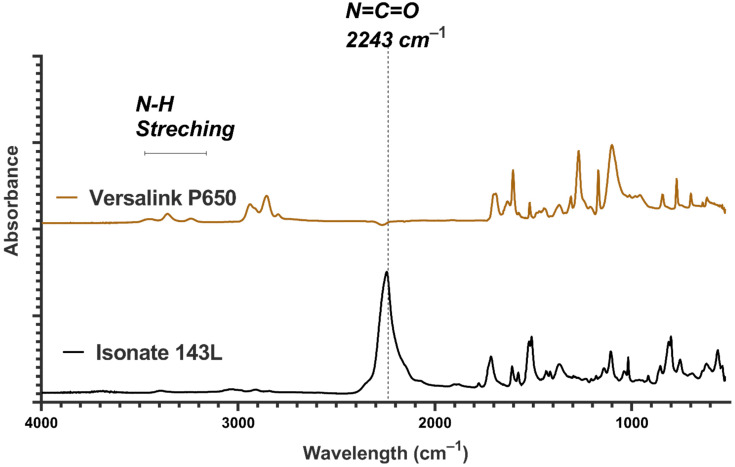
FTIR spectra of components A and B used for polyurea productions.

Figure 6 demonstrates the Fourier-transform infrared (FTIR) spectra for ISONATE 143L and Versalink P650 used to produce polyurea films. It could be noted that the N=C=O stretching band from isocyanate appeared at 2243 cm^−1^. Upon complete polymerization, the NCO band disappeared and urea linkages (O=C-(NH)_2_) formed.

**Figure 7:**
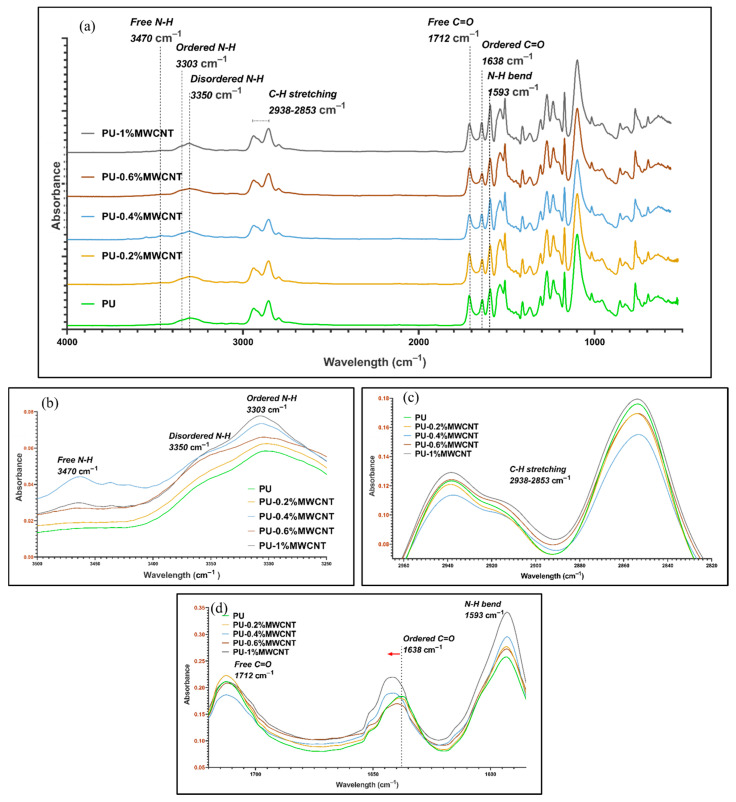
FTIR spectra of (**a**) PU and MWCNT nanocomposite films with loadings of 0.2%, 0.4%, 0.6%, and 1%, (**b**) N-H bands, (**c**) C-H bands, and (**d**) carbonyl bands.

**Figure 8:**
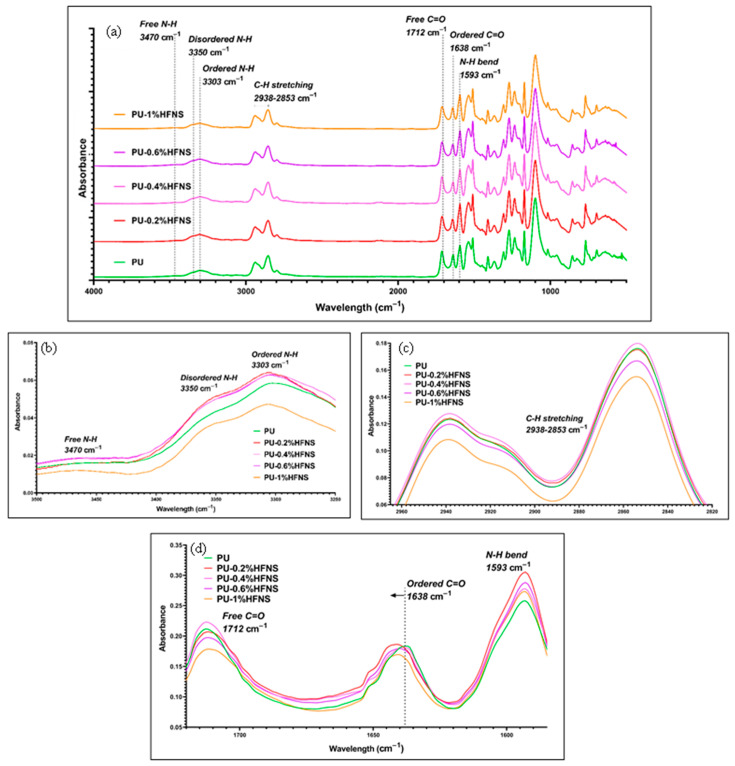
FTIR spectra of (**a**) PU and HFNS nanocomposites films with loadings of 0.2%, 0.4%, 0.6%, and 1%, (**b**) N-H bands, (**c**) C-H bands, and (**d**) carbonyl bands.

FTIR spectra for polyurea nanocomposite films are shown in Figure 7 and Figure 8, respectively. The disappearance of NCO bands at 2243 cm^−1^ in all the samples demonstrates that full polymerization occurred. The characteristics of polyurea with N-H stretching bands and C=O stretching bands at 3303–3470 cm^−1^ and 1638–1712 cm^−1^, respectively, could be observed. FTIR spectra demonstrated the presence of free, ordered, and disordered N-H stretching at 3470 cm^−1^, 3350 cm^−1^ and 3303 cm^−1^, respectively. Ordered stretching refers to bidentate H-bonded N-H in the hard domain with C=O, while disordered refers to the formation of monodentate H-bonds formed in the mixed-phase or amorphous state. Stretching bands of C=O bonds were also observed in ordered and free stretching at 1638 cm^−1^ and 1712 cm^−1^, respectively. The presence of free, disordered, and ordered stretching of those bonds confirmed two phases in the polyurea structure, i.e., hard and soft domains. The presence of only ordered C=O hydrogen bonds demonstrated a higher degree of microphase separation, as reported by He et al. [64]. A noticeable band at 1593 cm^−1^ demonstrated a bending of the N-H bond and, simultaneously, stretching of C=O bonds (amide II), confirming the presence of urea linkages and C-H bond stretching at 2853 cm^−1^ and 2938 cm^−1^, respectively. The spectrum of neat PU shows a similar band to research performed on the usage of ISONATE 143L and Versalink P650 by Castagna et al. [74] and on aromatic polyurea by Iqbal et al. [75].

Comparing individual bands for MWCNTs and HFNS from Figure 7 and Figure 8, respectively, it could be observed that for both MWCNTs and HFNS, there was a shift to the left for ordered C=O. This revealed that the addition of those two nanofillers disrupted the ordered arrangements of C=O in the hard domains and the diffusion of the soft segments into the hard domain. One can also note that the peaks for loadings of 0.4%, 0.6%, and 1% of MWCNT and HFNS nanocomposite films as compared to PU were broader, indicating a wider distribution of hydrogen bonds and, therefore, weaker hydrogen bonding within the hard domains [64]. The FTIR spectra for C=O bands correlated with the tensile results, whereby 0.2% of MWCNTs led to a higher tensile strength than the other loadings of MWCNTs and HFNS. Figure 7b shows that the addition of 1% of MWCNTs gave rise to free N-H bands, demonstrating a disruption by MWCNTs within the structure, preventing the formation of hydrogen bonds within the soft and hard domains. It can be noticed that the addition of 0.4% of MWCNTs seemed to be the starting point for agglomeration to occur, resulting in an inhomogeneous dispersion. This resulted in two phenomena: one was the chain wrapping on MWCNTs and the other was the formation of voids due to MWCNT aggregates between polyurea chains. The former induced H-bonding between the adjacent hard segments, while the latter created a barrier between polyurea chains, leaving free N-H bonds. This reasoning is in line with the slight decrease in tensile strength in PU/0.4%MWCNT from PU/0.2%MWCNT by only 7% and the reduced ductility confirmed by a 12% decrease in elongation. As for HFNS samples (Figure 8b), it could be observed that there were no peaks of free N-H bands. This could be due to the bonding of the silanol (Si-OH) groups present in the filler. The -OH might be bonding with the -NH group, creating hydrogen bonds, explaining the absence of free N-H bands in FTIR. This observation is in line with the decreasing tensile strength upon addition of HFNS. The hydrogen bond between Si-OH and -NH altered the spatial orientation and restricted crosslinking between urea groups within the matrix.

### 3.4. Degree of Hydrophobicity

**Figure 9:**
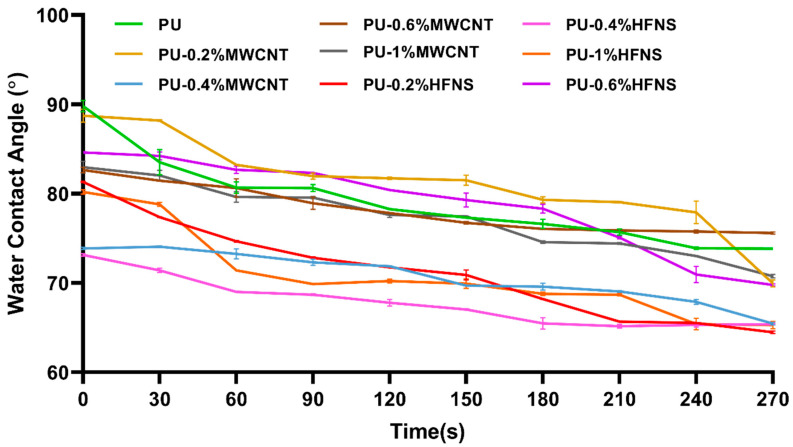
Water contact angle of PU and nanocomposite films from 0 s to 270 s.

**Figure 10:**
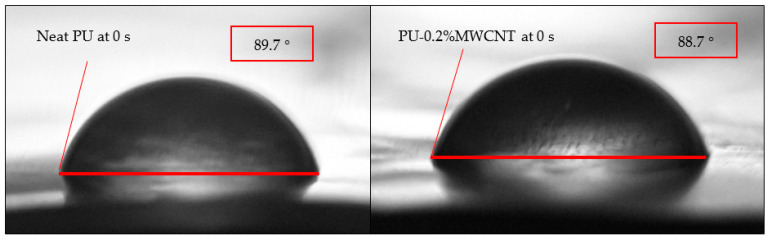
Drop appearance for neat PU and PU-0.2%MWCNT at 0 s during water contact angle experiments.

The water contact angle test measured the degree of hydrophobicity. Figure 9 shows the water contact angle for polyurea nanocomposites films over 270 s. As time increased, the contact angle decreased for all the samples, and the lowest contact angle was obtained for PU-0.4%HFNS. The highest contact angle of 89.7° was observed for PU, as seen in Figure 10. From the data obtained, it could be noted that MWCNTs and HFNS led to more hydrophilic behavior than PU. This can be explained due to the hydrophilic nature of HFNS, having an -OH group at the surface of the nanosphere and high surface free energy [76], causing a disruption of hydrogen bondings within the polyurea structure, as evidenced by FTIR spectra leading to a higher availability of polar bonds. MWCNTs are known to be hydrophobic with a water contact angle of around 156° [77]; however, the addition of MWCNTs compared to PU film did not increase the contact angle significantly. With a loading of 0.2% of MWCNTs, the water contact angle at 0 s was 88.7°, and the lack of increase in hydrophobicity could be due to the low loading of MWCNTs in the polymer matrix. A similar study with MWCNTs incorporated in acrylic resin showed that contact angles only showed a significant increase at 6 wt% [78]. Due to the high viscosity of polyurea, the addition of a higher loading of MWCNTs resulted in the solution being difficult to work on. However, as time progressed, the angle was higher than that of neat PU, with a sudden drop in water contact angle at 270 s. For other loadings, on the other hand, the water contact angles at 0 s up to 270 s were much lower than those of neat PU; therefore, MWCNT reinforcement affected the wettability of polyurea samples. However, the contact angle results did not describe much about the structure of the polyurea nanocomposites except that MWCNTs and HFNS may have increased the surface free energy that might arise from the disruption of hard domains and less strong hydrogen bonding as compared to neat PU [79]. The water contact angle for 0.2% of MWCNTs showed approximately a constant angle, indicating a retardation in water penetration and spread as compared to neat PU. As an anti-corrosive coating, the addition of only 0.2% of MWCNTs could reduce the corrosion rate and therefore elongate the lifetime of the metallic structures.

### 3.5. Surface Morphology of Aromatic Polyurea Membranes

**Figure 11:**
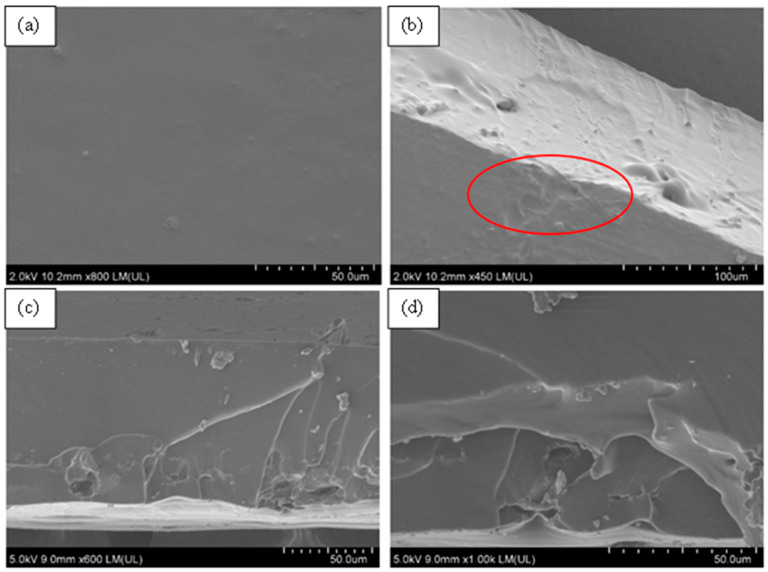
FE-SEM micrographs of neat PU. (**a**) Surface morphology at ×600, (**b**) side-view morphology at ×450, (**c**) tensile fracture surface ×600, and (**d**) tensile fracture surface at ×1000.

From Figure 11, the FE-SEM micrographs showed a solid smooth thin film with some roughness, which confirmed the production of solid aromatic polyurea thin films. Figure 11b demonstrates part of the cross-section of the film, whereby a very dense and solid film could be observed along with minor voids (air entrapment during spinning). The flow lines (circled) could be a result of the slow curing time of polyurea where fibers were rejoining together after deposition on the collector (Figure 1). As polyurea landed on the collector, the fibers were still in gel form. Therefore, it was affected by the centrifugal force-driven flow on the collection surface, allowing them to merge and create a nonporous film [80,81]. Figure 11c,d show the fracture surface of PU. Figure 11d shows some delamination of the polyurea layers produced by electrospinning. This could be an indication of ductile drawing, which is supported by the tensile elongation of 360% [82]. The smooth fracture surface is also an indication of ductile fracture of the polyurea films [83].

**Figure 12:**
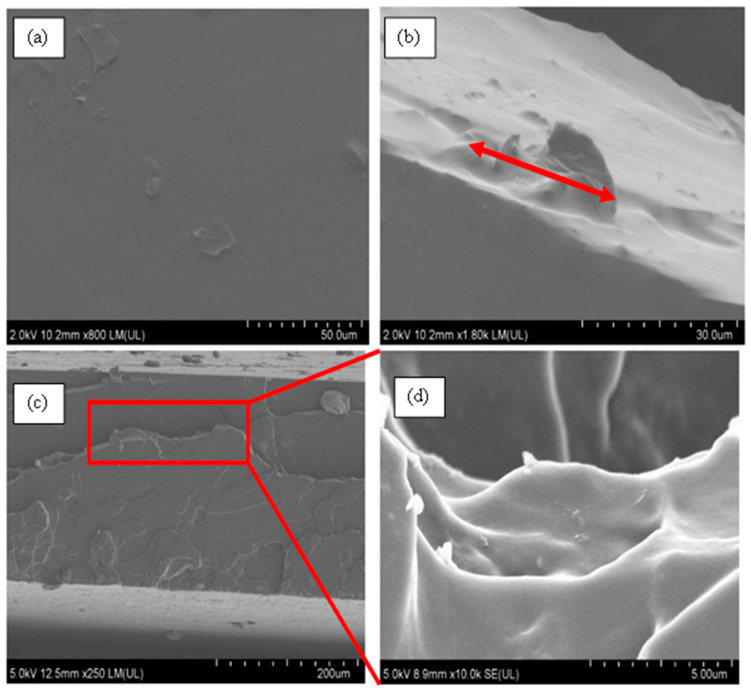
FE-SEM micrographs of PU-0.2%MWCNT. (**a**) Surface morphology at ×800, (**b**) cross-sectional morphology prior to fracture, (**c**) tensile fracture surface at ×250, and (**d**) fracture lines on fractured surface at ×10k.

From Figure 12b, a minor agglomeration of size 24 μm (red arrow) of MWCNTs could be observed on the cross-section of the film, while the majority area of the film showed a dense, uniform, and solid structure at other sites. A similar observation was made by Wang et al. [84]. In some cases, there were rows of cavitation within the MWCNT sample and straight crazing lines (Figure S2 in Supplementary Materials). These were caused by the electrospinning that stretched the fibers, overcoming the surface tension creating fibers. Due to the medium curing time of polyurea, those fibers flowed on neighboring fibers closing the pores. The lines could be attributed to the high electrical conductivity of MWCNTs, therefore increasing the polymer solution’s stretching.

Figure 12c,d display the fracture surface of PU-0.2%MWCNT. Figure 12c exhibits multiple fracture lines, which could indicate a ductile fracture. The fracture lines observed in Figure 12d demonstrate that MWCNTs at low loading in PU may have provided a higher resistance to strain hardening, which gave rise to its high strength.

**Figure 13:**
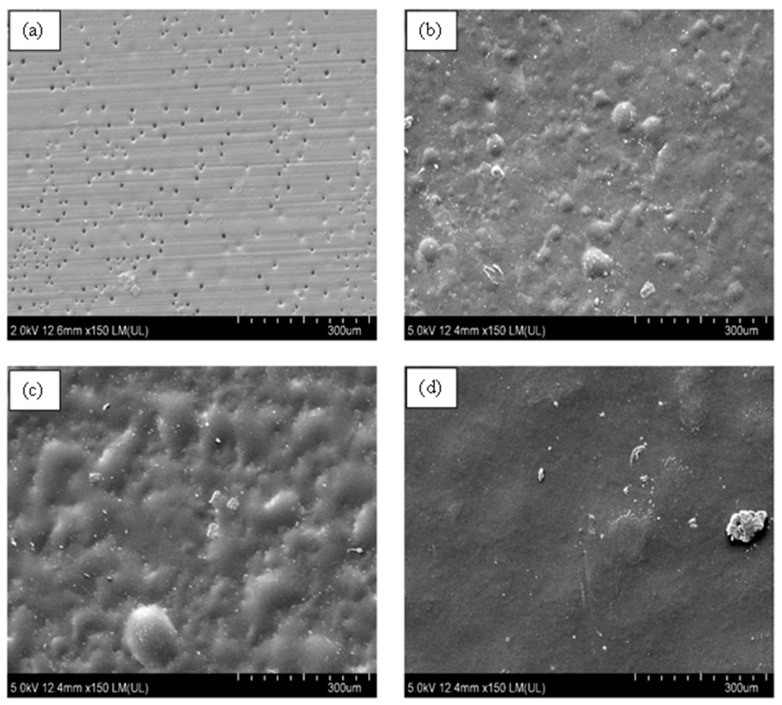
PU surface at magnification of ×150 with (**a**) 0.2% MWCNTs, (**b**) 0.4% MWCNTs, (**c**) 0.6% MWCNTs, and (**d**) 1% MWCNTs.

Figure 13 demonstrates the surface morphology of PU with loadings of 0.2%, 0.4%, 0.6%, and 1% of MWCNTs. It could be observed that as the loading increased from 0.2% to 0.4% and 0.6% (Figure 13b,c), the surface pores disappeared, and bumps were formed on the sample. At a loading of 1% (Figure 13d), the surface became completely void-free (at the surface) with some bumps; this could be due to the agglomeration of MWCNTs within the sample, repelling water molecules as they deposited on the collector. The hydrophobic nature of MWCNTs reduced the condensation of the water droplets due to high humidity in air, resulting in the disappearance of surface pores at a loading of 1%. From Figure 13a, it could be observed that aligned pits of around 7 μm (Figure S2b in Supplementary Materials) were present on the surface because of evaporation of water vapor around the MWCNTs. Casper et al. [85] described this phenomenon as breath figures, whereby evaporation occurs as the jets are propelled to the collector. As the surface of the jet cools down due to heat release because of evaporation, water condenses, and as the samples dry, the water droplets leave an imprint. The formation of pores is most likely due to the high humidity in the environment. The alignment of these pores could prove the production of polyurea fibers that merge due to the gel-state of polyurea upon deposition on the collector. This phenomenon could be eradicated by controlling the humidity level to lower the percentage of water vapor in the air. This could be achieved by incorporating a nitrogen purge in the electrospinning process prior to the coating or by applying heat to the metal surfaces to force the water vapor out of the surface prior to curing. The morphology and structural property of thin films obtained in this study were compared to the result reported by Tripathi et al. [65] with respect to the concentration of polyurea in the solvent and the curing time of polyurea herein. The Versalink P650 used in this study is a larger-molecular-weight amine contributing to higher soft segmental dynamics. MWCNTs are known to be immiscible in water and hydrophobic, allowing the water droplets to remain on the film’s top surface, leading to the creation of pores upon evaporation [86].

**Figure 14:**
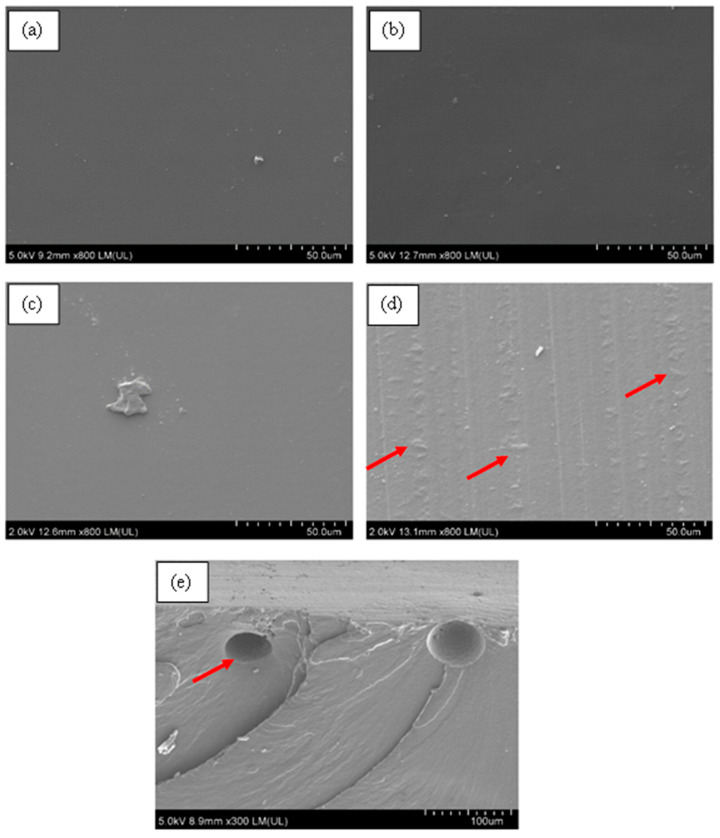
PU at magnification ×800 with (**a**) 0.2% HFNS, (**b**) 0.4% HFNS, (**c**) 0.6% HFNS, (**d**) 1% HFNS, and (**e**) fracture surface of PU-0.4% HFNS at magnification of ×300.

Figure 14a–d show that as the loading increased to 1%, some lines were visible with an agglomeration of HFNS particles. The horizontal streaks in Figure 13a and the vertical streaks in Figure 14d were the result of the merging of fibers deposited during the electrospinning process. This occurs when there are irregularities on the surface; therefore, the merging does not occur smoothly, due to the obstructions in between. It is important to note that the orientation of the streaks is due to the position of the samples viewed under the FE-SEM and is not dependent on the processing conditions. Figure 14e demonstrates the tensile fracture surface at a 0.4% HFNS loading; the fracture lines appeared sparse with a relatively great distance compared to 0.2% of MWCNTs, and there were river-like lines originating from the cavities due to high stress concentrations. The formation of those defects from the cavities demonstrated that the fracture emerged from the voids and travelled throughout the cross-section of the polyurea [82]. This could be the result of the lower tensile and elongation of HFNS. The formation of voids is an indication of the agglomeration of HFNS within the polyurea matrix. A similar observation was reported by Pakula et al. [87].

### 3.6. Modulated Thermogravimetry (MTGA)

**Figure 15:**
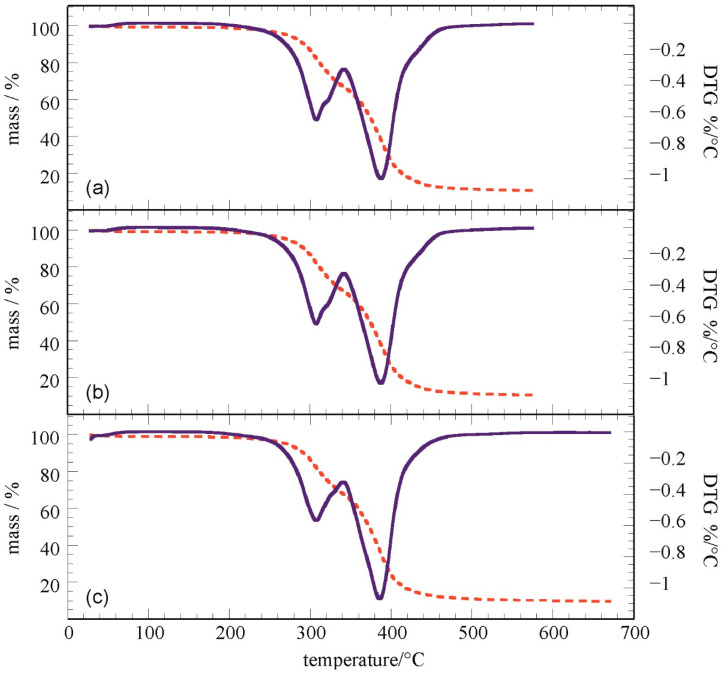
Thermogravimetric curves for (**a**) PU, (**b**) PU-0.2%MWCNT, and (**c**) PU-0.4%HFNS films.

Thermogravimetric data evidenced the presence of a two-step degradation mechanism for all samples. It is widely reported that the thermal degradation of polyurea is related to the microphase-separated morphology that consists of hard segment domains dispersed in a matrix consisting of segments [88,89]. The hard domains are extensively hydrogen-bonded, and they provide toughness to the material. Based on the literature assignment [88], the hard segment has a lower thermal stability than the soft segment. This is due to the urea groups (from the hard domains) that enhance thermal volatilization as they contain oxygen atoms. At this degradation temperature, the urea bond breaks down into isocyanate and diamine. The composites showed a similar degradation profile to that of pure polymer shown in Figure 15, with a first degradation step representing 15% of the total mass loss. Concerning this, the temperature at the maximum degradation rate for each step showed a slight increase in both temperature values for the composites.

From Table 1, it is understood that a 0.2% MWCNT loading in polyurea was sufficient to give a similar degradation temperature increase to a 0.4% HFNS loading. MWCNTs at a 0.2% loading provided a better insulating layer to polyurea during thermal degradation than a 0.4% HFNS loading did. This could be attributed to the superior thermal performance of carbon nanotubes in general and the stronger intermolecular forces between them and polyurea chains. HFNS at a 0.4% loading may have contributed to agglomeration and weakening of the intermolecular forces with polyurea chains. To give more insight into the thermal stability of the composites, the kinetics parameters such as the activation energy for the degradation process is a more robust parameter [90,91]. Therefore, MTGA results were analyzed through real-time deconvolution techniques (discrete Fourier transformation) to obtain the activation energy values (E_a_) as functions of the extent of conversion without an assumption on the degradation model [92]. The obtained E_a_ values are provided in Table 1, and they demonstrate the increase in the thermal stability of the polymer in the composite material. It should be noted that within the experimental errors, the two composites had the same thermal stability. One can conclude that the fillers generated a thermal stabilization of the polymer without significantly altering the ratio between hard and soft domains in the matrix.

### 3.7. Viscoelastic Properties Polyurea Nanocomposites Membranes

Dynamic mechanical analysis (DMA) experiments investigated the effects of the fillers (MWCNTs and HFNS) on the viscoelastic behavior of the polyurea aromatic films. As highlighted in Table 2, the rheological parameters at 25 °C were affected by the addition of both fillers within the polymeric matrix. It was observed that both storage (G’) and loss (G”) moduli increased in the nanocomposite materials. The tan(δ) values (calculated by the G’’/G’ ratios) evidenced that the viscous component was enhanced by the presence of both MWCNTs and HFNS in the PU matrix.

**Figure 16:**
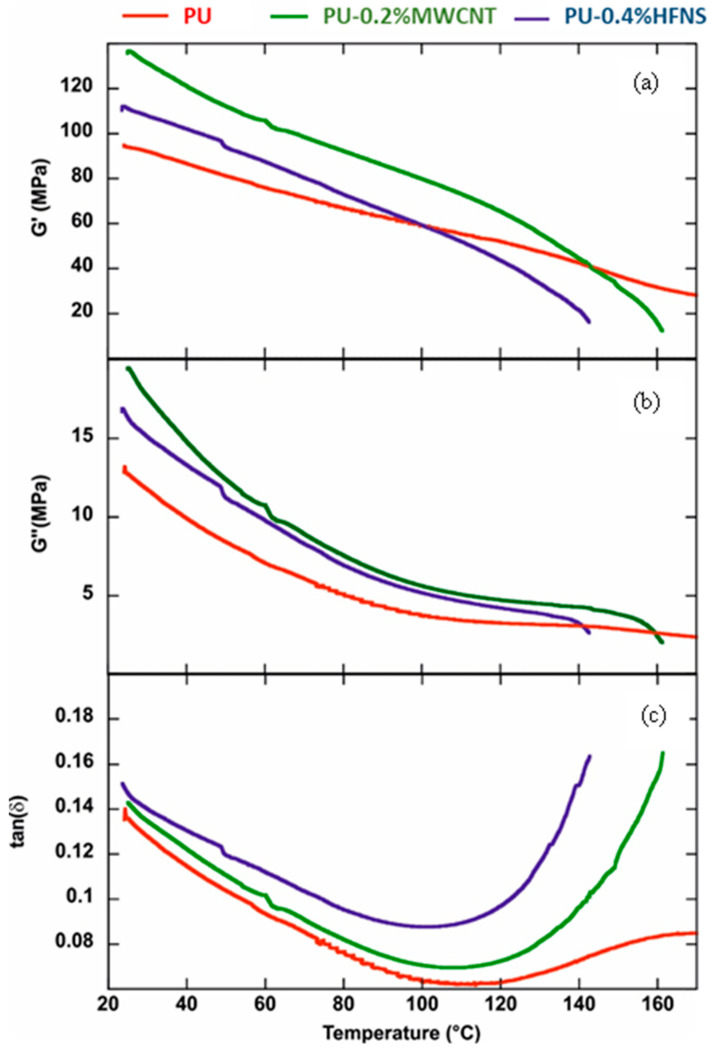
(**a**) Storage modulus, (**b**) loss modulus, and (**c**) tan (δ) as functions of temperature for PU, PU-0.2%MWCNT, and PU-0.4%HFNS films.

Figure 16 shows the influence of the temperature on the rheological parameters of PU-based films. As a general result, we detected that both rheological moduli decreased with the temperature within the investigated range (Figure 16a,b). On the other hand, the tan(δ) vs. temperature plots presented two regions: (1) decreasing trends from 25 to ca. 100 °C, indicating that larger temperatures improve the films’ energy storage capacity; (2) sudden tan(δ) rises due to the further increase in the temperature. According to the literature [93], the tan(δ) increase at a temperature higher than 100 °C could be attributed to the order–disorder transition of polyurea. It was observed that the presence of HFNS filler did not alter the PU transition temperature, which was determined by the onset point in the range of 100–150 °C. Precisely, the onset temperatures of 124.1 °C and 122.5 °C for PU and PU-0.4%HFNS films were calculated, respectively. Contrarily, the addition of MWCNTs generated a slight increase in the onset temperature (132.3 °C).

## 4. Prospects of Electrospun Polyurea Films as Anti-Corrosive Coatings

The integration of flexibility and corrosion protection functionalities in polymeric matrices is rewarding for diverse end-user and industrial applications such as heat exchangers, metallic tubes, and electrical cables and devices. Internal tubes of heat exchangers are often in contact with corrosive fluids such as water vapor condensate [94], leading to the leaching of metallic particles into the fluid [95]. Furthermore, heat exchangers operate at high temperatures and pressure, causing thermal expansion of the tubes; thus, external coatings applied on the metallic tube surface must be corrosion-resistant and withstand high thermal expansion, retaining their mechanical properties. Similarly, wearable devices, energy harvesters, and thin-film sensors also require flexible and anti-corrosive coatings as a barrier protection for liquid or water vapor to protect the electronics [96].

While studies of such thin and anti-corrosive coatings exist in the literature, as shown in Table 3, their stretchability has rarely exceeded 5% tensile strain or, in some cases, the results have seldom been communicated. For example, the epoxy coatings described by Sung et al. [97] and Kumar et al. [98] showed a low elongation of only 3.7% and 1.1%, respectively, demonstrating the lack of bendability and flexibility. Zhang et al. [99] studied a combination of PDMS and TFB to achieve a flexible and hydrophobic coating for stainless steel. They found that the elongation was around 700% at a 5 mm/min stretching speed and 150% at 20 mm/min. This demonstrates that as the stretching speed increased, the elongation drastically decreased. On the other hand, the nonporous films developed in our study demonstrated an elongation beyond 350% at 500 mm/min, which favors its application as a flexible coating for metallic plates or tubular structures subjected to sudden and impulsive impact. The fabrication method of flexible thin films is a crucial factor in determining defect-free and uniform coatings. The casting and spray-coating methods used by the studies in Table 3 have potential downsides. For example, casted polymeric coatings could lead to uneven thickness, causing stress concentration and, therefore, crack formation [100]. Spray coating, on the other hand, deposits microdroplets, leading to the formation of pinhole defects [53]; therefore, it allows oxygen and chloride ions to attain the metal and consequently accelerate the corrosion process. As compared to these methods, the electrospinning or electrospraying technique is a comprehensive way to deposit a thin polymeric layer on the metallic structures, especially those complex structures. This is mainly because the thickness of the layer can be easily controlled by the solutions’ flow rate and time of exposure to the high voltage.

The polyurea films in this study exhibited excellent viscoelastic properties with a storage modulus ranging from 94 MPa to 136 MPa, high mechanical strength (14–21 MPa), significantly high elongation at break (360–400%), and water contact angle of nearly 90°. It can be considered as a good candidate as an ultra-flexible anti-corrosion film for tubes of heat exchanges or flexible electronics. Upon comparison, the electro-spun polyurea coating showed a similar trend for different properties such as tensile strength, thermal stability distinguished from degradation temperature and activation energy, and the degree of hydrophobicity. However, polyurea films demonstrated a higher flexibility and viscoelasticity as compared to the other coatings, demonstrating its potential as a thermally stable, nonporous, and flexible coating for metal to defend against corrosion. Further studies such as Electrochemical Impedance Spectroscopy (EIS) or weight loss in a corrosive environment could be performed to enhance the understanding of the degree of protection polyurea has.

## 5. Conclusions

This study successfully fabricated nonporous polyurea membranes of a thickness of around 200 μm and above. The tensile test analysis showed that a low loading of multi-walled carbon nanotubes (MWCNTs) improved the strength of polyurea films due to the wrapping of the polyurea chain around the carbon nanotubes. The addition of hydrophilic fumed nanosilica (HFNS) spheres did not improve the strength, due to the disruption of the polyurea chains hindering the intrinsic bonding of urea linkages. Chemical interaction for both nanofillers demonstrated a shift in the C=O band. This indicates a disruption in the ordered arrangements of C=O in the hard domains and diffusion of the soft segments into the hard domain. The surface of the neat polyurea sample was smooth with the merging of fibers upon curing. At a loading of 0.2%, MWCNTs manifested the formation of aligned pores due to water evaporation settled from vapor in the surroundings, leaving an imprint on the surface. The thermal degradation temperature increased with the addition of nanofillers, therefore increasing the thermal stability of polyurea. Dynamic mechanical analysis showed an increase in the storage and loss moduli on the addition of nanofillers and an increase in the viscous component. HFNS did not change the order–disorder transition temperature of polyurea, while MWCNTs demonstrated a slight increase. The high mechanical strength, optical transparency, hydrophobicity, and the smooth surface of thin-film polyurea nanocomposite membranes achieved a robust, nonporous, and flexible thin film with potential application as an anticorrosion coating for metallic tubes in heat exchangers, electrical cables, or flexible electronics containing metallic parts.

## Figures and Tables

**Table 1:** Thermal degradation parameters from MTGA experiments.

Film	T_d1_ (°C)	T_d2_ (°C)	E_a_ (kJ mol^−1^)
PU	302.1	380.2	160 ± 9
PU-0.2%MWCNT	307.7	387.4	182 ± 8
PU-0.4%HFNS	307.7	386.1	187 ± 9

**Table 2:** Rheological parameters of PU-based films at 25 °C.

Film	Storage Modulus (MPa)	Loss Modulus (MPa)	tan(δ)
PU	93.9	12.75	0.135
PU-0.2%MWCNT	135.9	19.43	0.143
PU-0.4%HFNS	111.2	16.35	0.147

**Table 3:** Comparison between some previous research on anti-corrosive coatings and this study.

Reference	Sung et al. [97]	Kumar et al. [98]	Yanhai et al. [101]	Zhu et al. [102]	Zhang et al. [99]	Our Study
Metal	-	Mild steel	Mild steel (1015)	Tin	Stainless steel	-
Polymer blend	3,4-epoxycyclohexylmethyl-3,4-epoxycyclohexanecarboxylate (ECHM) and Dihydroxyl Soybean Oil blend (DSO)	MWCNT/Epoxy resin (Cam coat 2071)	Polytetrafluoroethylene in Ni-Cu-P coating	Bisphenol A-based benzoxazine (BA-a), polyurethane, and mesoporous SiO_2_ (SBA-15)	bis(amine)-terminated poly(dimethylsiloxane) (H2N-PDMS-NH2) and 1,3,5-triformylbenzene (TFB)	Aromatic polyurea
Deposition method	Casting	Spray coating	Electroless plating	Spray coating	Casting	Electrospinning
Tensile Strength	34.5 MPa (After 10 days)	~75 MPa (with 0.75 wt% MWCNT)	-	-	0.035 MPa	14.1 MPa(Neat) 20.8 MPa (0.2 wt% MWCNT)
Elongation	3.7%	1.1%	-	-	150% (20 mm/min)	360% (Neat) 402% (0.2 wt% MWCNT) (500 mm/min)
Water contact angle	-	-	-	150°	123.2°	90°
Degradation temperature	428 °C (50% mass loss)	342 °C (10% mass loss)	-	-	Stable until 525 °C	15% mass loss Neat: 302.1 °C 0.2% MWCNT: 307.7 °C
Activation energy (kJ/mol)	-	-	290	149.3	-	Neat: 160 0.2% MWCNT: 182 0.4% HFNS: 187
Transparency	90%	-	-	-	80%	Neat PU: Optically transparent
Corrosion resistance before coating	-	-	1.75 mg/cm^2^ mass loss (7 days in 3.5% NaCl solution)	2.14 × 10^−4^ A cm^−2^ corrosion current density (10 days in 3.5% NaCl solution)	-	-
Corrosion resistance after coating	-	99.99% protection efficiency (0.75 wt% MWCNT) in 3.5% NaCl solution.	0.1 mg/cm^2^ mass loss (7 days in 3.5% NaCl solution)	8.9 × 10^−5^ A cm^−2^ corrosion current density (10 days in 3.5% NaCl solution)	Contact angle showed no significant change 8 days in 4% NaCl	-
Targeted application	Coating applications	General anti-corrosion coating	Heat exchanger	Superhydrophobic surfaces	Anti-corrosion coating and flexible electronics	General coating for anti-corrosion

## Data Availability

The raw/processed data required to reproduce these findings cannot be shared at this time, as the data also form part of an ongoing study.

## Supplementary Materials

The following are available online at https://www.mdpi.com/article/10.3390/nano11112998/s1, Figure S1: Tensile Sample coupon, Table S1: Dunett’s Comparison test for tensile strength of polyurea nanocomposites films, Table S2: Dunett’s Comparison test for maximum elongation of polyurea nanocomposites films, Figure S2: FESEM of PU-0.2% MWCNT (a) Surface morphology at ×350 magnification, (b) Size of one of the pores at ×4.50 k magnification.
