# Supplementary material for: Nonporous, Strong, Stretchable, and Transparent Electrospun Aromatic Polyurea Nanocomposites as Potential Anticorrosion Coating Films
Source: Nanomaterials (Basel). 2021 Nov 8;11(11):2998. doi: 10.3390/nano11112998 (PMC8618329; doi:10.3390/nano11112998)
Supplement: Supplementary file 1 [file nanomaterials-11-02998-s001.zip › nanomaterials-1439113-supplementary.pdf]

Supplementary Information

Nonporous, Strong, Stretchable, and Transparent Electrospun Aromatic Polyurea Nanocomposites as Potential Anticorrosion Coating Films

Sheik Ambarine Banon Auckloo [1], Khanisya Palaniandy [1], Yew Mun Hung [1], Giuseppe Lazzara [2], Siang-Piao Chai [3] and Pooria Pasbakhsh [1,*]

[1] Mechanical Engineering Discipline, School of Engineering, Monash University Malaysia, Jalan, Lagoon Selatan, Bandar Sunway, Subang Jaya 47500, Malaysia; sheik.auckloo@monash.edu (S.A.B.A.); khanisya.palaniandy1@monash.edu (K.P.); hung.yew.mun@monash.edu (Y.M.H.)
[2] Department of Physics and Chemistry, University of Palermo, Viale delle Scienze, pad. 17, 90128 Palermo, Italy; giuseppe.lazzara@unipa.it
[3] Multidisciplinary Platform of Advanced Engineering, Chemical Engineering Discipline, School of Engineering, Monash University Malaysia, Jalan Lagoon Selatan, Bandar Sunway, Subang Jaya 47500, Malaysia; chai.siang.piao@monash.edu
* Correspondence: pooria.pasbakhsh@monash.edu; Tel.: +60-3551-46211; Fax: +60-3551-46207

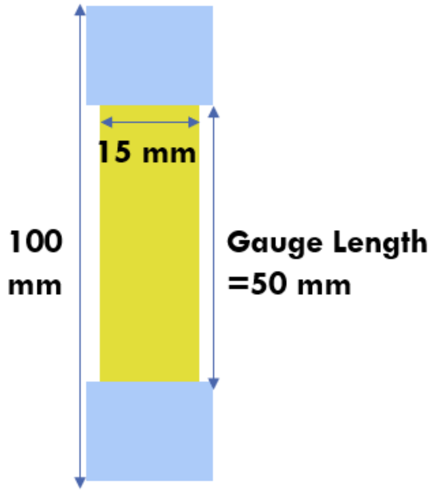

Figure S1. Tensile Sample coupon.

Table S1. Dunett's Comparison test for tensile strength of polyurea nanocomposites films.

Dunnett's multiple comparisons test	Mean Diff.	Adjusted P Value
PU vs. PU-0.2%MWCNT	-6.761	<.001
PU vs. PU-0.4%MWCNT	-5.232	<.001
PU vs. PU-0.6%MWCNT	-0.1515	>.999
PU vs. PU-1%MWCNT	3.338	.009
PU vs. PU-0.2%HFNS	0.08516	>.999
PU vs. PU-0.4%HFNS	-1.783	.268
PU vs. PU-0.6%HFNS	5.694	<.001
PU vs. PU-1%HFNS	7.403	<.001

Table S2. Dunett's Comparison test for maximum elongation of polyurea nanocomposites films.

Dunnett's multiple comparisons test	Mean Diff.	Adjusted P Value
PU vs. PU-0.2%MWCNT	-42.33	.143
PU vs. PU-0.4%MWCNT	8.533	.997
PU vs. PU-0.6%MWCNT	39.43	.191
PU vs. PU-1%MWCNT	207.9	<.001
PU vs. PU-0.2%HFNS	93.63	<.001
PU vs. PU-0.4%HFNS	61.90	.016
PU vs. PU-0.6%HFNS	221.8	<.001
PU vs. PU-1%HFNS	288.0	<.001

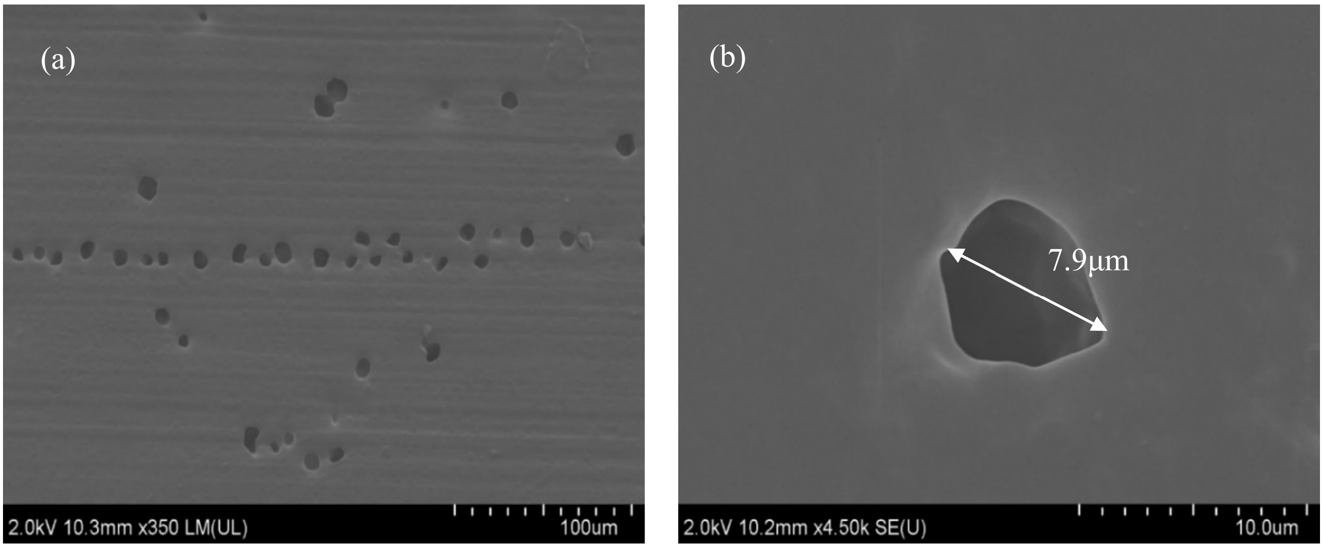

Figure S2. FESEM of PU-0.2% MWCNT (a) Surface morphology at x350 magnification, (b) Size of one of the pores at x 4.50k magnification.
